# The Motivational Salience of Infant Faces Is Similar for Men and Women

**DOI:** 10.1371/journal.pone.0020632

**Published:** 2011-05-31

**Authors:** Christine E. Parsons, Katherine S. Young, Nina Kumari, Alan Stein, Morten L. Kringelbach

**Affiliations:** 1 University Department of Psychiatry, University of Oxford, Oxford, United Kingdom; 2 Center of Functionally Integrative Neuroscience, Aarhus University, Aahrus, Denmark; The University of Hong Kong, Hong Kong

## Abstract

Infant facial features are thought to be powerful elicitors of caregiving behaviour. It has been widely assumed that men and women respond in different ways to those features, such as a large forehead and eyes and round protruding cheeks, colloquially described as ‘cute’. We investigated experimentally potential differences using measures of both conscious appraisal (‘liking’) and behavioural responsivity (‘wanting’) to real world infant and adult faces in 71 non-parents. Overall, women gave significantly higher ‘liking’ ratings for infant faces (but not adult faces) compared to men. However, this difference was not seen in the ‘wanting’ task, where we measured the willingness of men and women to key-press to increase or decrease viewing duration of an infant face. Further analysis of sensitivity to cuteness, categorising infants by degree of infantile features, revealed that both men and women showed a graded significant increase in both positive attractiveness ratings and viewing times to the ‘cutest’ infants. We suggest that infant faces may have similar motivational salience to men and women, despite gender idiosyncrasies in their conscious appraisal.

## Introduction

Adults are remarkably attuned to the facial features that characterise their young, such as a large rounded forehead, large low-set eyes, a short and narrow nose and a small chin [1], [2], [3]. Lorenz [4], [5] argued that humans have a natural attraction to these features and that such an attraction evolved to enhance motivation to engage in caregiving behaviour. We have recently identified a putative neural signature of this ‘parental instinct’ [6]. In species, such as humans, whose young depend so heavily on the early caregiver-infant relationship [7], this attraction is likely to enhance offspring survival and development [8], [9], [10], [11]. Within this conceptualisation, cuteness is a configuration of visual features that has a specific biological function-promotion of infant nurturance.

Adults’ typical initial response to an infant picture is a smile [12]. Both children and adults consistently prefer pictures of infants over pictures of adults [13], [14]. Infants are the object of a variety of other nurturing and affectionate impulses, such as high-pitched vocalisations (i.e. “motherese” [15]), preferential looking [16], leniency [17], and protectiveness [2]. This disposition to respond positively to infantile features is intricately linked to caregiving behaviour. Yet, little is known about the nature of perception of the physical properties of a ‘cute’ infant face, and how this shapes our immediate behaviour.

The ability to perceive subtle differences in infant attractiveness has been the focus of some recent work. Women have been shown to be slightly better than men at detecting gradations in manipulated cuteness in infant faces [18], despite equal performance in detecting emotional valence and age differences [19]. Women have long been credited with having a greater interest in infants and greater skill in interacting with them, e.g., [20], but gender differences in responding to the young are far from clear cut (see [21] for a review). Some studies have reported that women are generally more perceptive and responsive to cuteness than are men (e.g., they smile more at a cute infant, [12]), but these effects have been found to vary across the lifespan, e.g. [22]. One study reported that preference for infantile head shapes was more pronounced in women than in men [3], while another did not [23]. Given these discrepancies, and the increasing acknowledgment of men’s role in nurturing their infants (e.g., [24]) investigation of both men and women’s responses to infant faces is warranted.

Adults might be adept at perceiving subtle differences in infant facial configuration, but the question arises, do these differences actually impact upon their behaviour? The predominant behavioural paradigms in the investigation of facial features and cuteness have required participants to consciously rate the attractiveness of infant faces, or make a choice between two. Such paradigms do not tap into the recent scientific progress in understanding the sub-components underlying the evaluation of hedonic stimuli, which has been demonstrated to consist of at least three components, including hedonic appraisal (‘liking’), incentive salience (‘wanting’) and learning, subserved by partially separable neural mechanisms [25], [26]. We therefore asked whether, beyond simple appraisal, viewing images of infant faces could shape immediate behaviour in an experimental paradigm. In addition to a ‘liking’ task measuring the conscious appraisal, we used a key press ‘wanting’ task to examine the amount of work participants would perform in order to change the relative duration for which they viewed an individual image (see [27], [28], [29]).

We asked whether differences in facial structure are salient when adults respond to ‘real world’, healthy infants falling within the natural occurring range of attractiveness. This is in contrast to recent studies which have used morphed infant faces where specific features have been modified to systematically increase or decrease attractiveness (e.g. [30], [31]). The use of these morphed images limits the external validity of studies as differences between images do not reflect natural variation in ‘cuteness’ [32], [33].

In order to test whether there is something specific about the way adults respond to infant faces, we also compared men and women’s responses to a set of adult faces. To investigate general responsivity to infants rather than to specifically one’s own infant, we chose to test a population of participants with little experience of caring for young infants.

## Materials and Methods

### Ethics Statement

The experimental procedures were approved by the Oxfordshire Research Ethics Committee B (12/07/2010). Participation was voluntary, and written consent was obtained prior to participation.

### Stimuli

Stimuli consisted of a total of 70 images of infant and adult faces (35 of each). The adult stimuli consisted of 18 images of females and 17 images of males. The infant images were obtained from a standardised database described elsewhere [6] and parental permission was obtained for the use of these images. The use of these images for research purposes was also approved by the Oxford Research Ethics Committee. The adult face images were obtained from several standardised databases [http://pics.psych.stir.ac.uk, 34,35]. All faces were previously rated as showing a neutral expression and were forward facing with comparable direction of eye gaze. In order to use as homogenous a sample of adult images as possible, images of adults of average attractiveness were. All images were presented in grayscale and were matched for size and luminosity. Participants viewed the faces on a computer monitor, such that face stimuli subtended a visual angle of approximately 4×2 degrees.

### Participants

A sample of 71 healthy participants with little or no experience of caring for young infants took part in this study with informed consent. Thirty-four of the participants were male and 37 female, with an age range of between 17 and 24 years (M = 20.05, SD = 1.45).

### Procedure

We used two measures, a ‘liking’ and a ‘wanting’ task, to capture the dual aspects of appraisal and incentive salience in adults’ hedonic processing of infant and adult faces. The appraisal task required participants to rate the attractiveness of the faces (“You are going to see a series of faces. Your task is to rate how attractive you find each picture.”). This provided a measure of ‘subjective liking’ of the images, similar to the task we have used extensively for measuring ‘liking’ of other hedonic stimuli, [e.g. 36]. The word ‘attractive’ was used based on several considerations. First, we wished to directly compare participants’ ‘liking’ ratings of adults and infants. Using different terms is potentially problematic in this regard. Second, the term ‘attractive’ has been used in a number of previous studies of adults’ responses to infant faces [37], [38], [39], [40]. Third, an independent panel of ten adults rated a subset of the infant faces on two scales: ‘cuteness’ and ‘attractiveness’; ratings were highly correlated (**r_s_** = 0.83, p<.0001).

The ‘wanting’ or ‘key-press’ task required participants to key-press to either increase or decrease the relative viewing duration of each image (“You are going to see a series of faces. In this task, you can control how long you view each image for.”). This task probed the incentive salience or ‘wanting’ to view the faces by measuring the amount of work participants are willing to do (and the resultant viewing times) in response to each stimulus, which in some respects was similar to other key-pressing tasks [29], [40], [41], [42].

**Figure 1:**
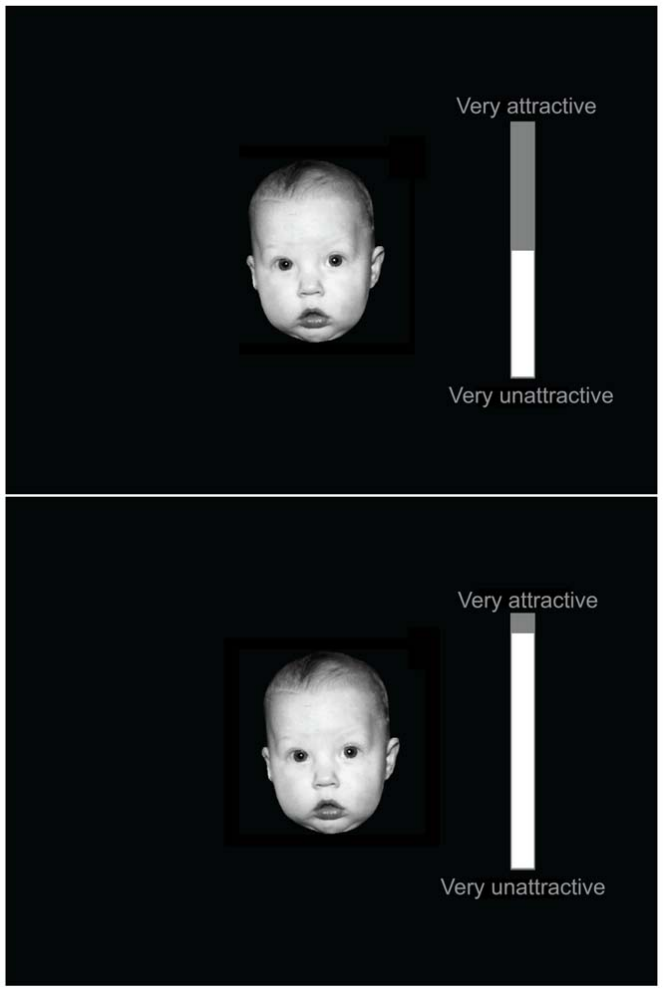
Screenshots of the ‘liking’ task. Participants were initially presented with a face image and a visual analogue scale (left) and were given 5 seconds to rate the image (right). The ‘wanting’ task was visually similar, except that the labels ‘very attractive’ and ‘very unattractive’ were absent, and the height of the white bar of the visual analogue scale decreased over time (the speed of this movement could be either increased or decreased by key-pressing).

In both tasks the participants were presented with a face image on the centre of the screen and a vertical visual analogue scale immediately to the right (see Figure 1). In the ‘liking’ task, participants were asked to rate the attractiveness of images of infant and adult faces using a visual analogue scale. Responses on this scale were measured from +4 ‘Very attractive’ to −4 ‘Very unattractive’. Participants made their rating by using the ‘up’ and ‘down’ keys to adjust the bar. Each stimulus was presented for five seconds and participants rated the 70 stimuli twice each. The order of stimuli was pseudorandomised across participants, by creating four versions of the task with different stimuli orders in each version. Ten participants completed each version. The order of completion of the ‘wanting’ and ‘liking’ task was also counterbalanced across participants.

In the ‘wanting’ task, the default viewing time of each stimulus was 5 seconds and participants could adjust this viewing time according to their ‘work-effort’, i.e. the frequency of key-pressing of either the ‘up’ or the ‘down’ keys. The visual analogue scale again presented on the right of each stimulus provided participants with a real time indication of the viewing time duration similar to an egg timer, with a bar moving downwards over time (the speed of movement could either be slowed or increased by the key-presses). Participants were also told that the key-press task would last for a set duration, independent of their responses. In both tasks, participants responded using the index finger of their dominant hand.

In order to investigate the effects of differences in facial feature configurations on infant cuteness/attractiveness ratings, we measured various dimensions of the infant faces, following the procedure described by Glocker et al. [31]. We measured the length and width of the whole face, as well as the size of individual facial features (namely the length and width of the nose, length and width of the eyes, mouth width and forehead length). In addition to Glocker et al.’s method, we included a measure of eye height in order to obtain a more complete measure of eye size. All measures were calculated as proportional indices relative to overall face width or length (i.e. nose length/face length, nose width/face width, eye length/face length, eye width/face width, mouth width/face width and forehead length/face length). Z-scores of these measures were used to quantify the extent of the ‘infantile features’ in each face. Infant faces were then divided into three groups: high infantile features, average infantile features, low infantile features, taken to reflect ‘cuteness’.

## Results

**Figure 2:**
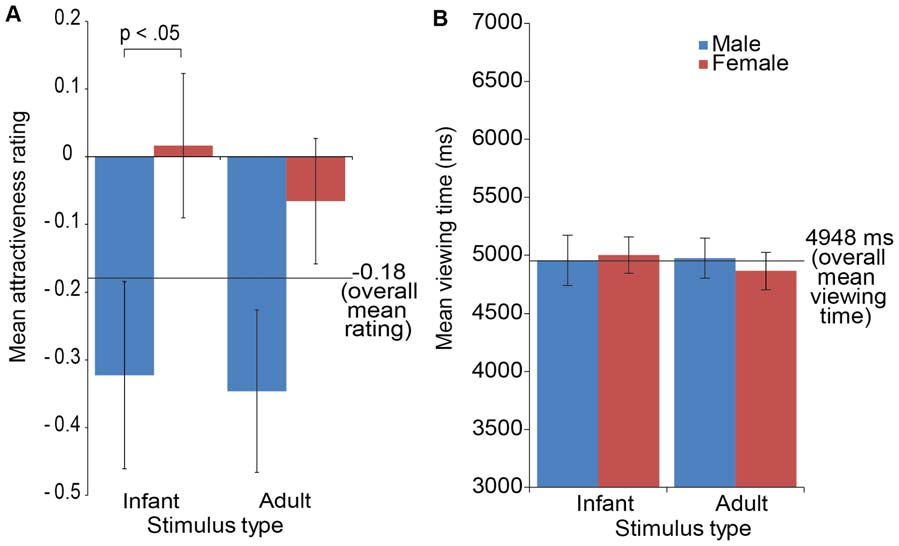
‘Liking’ and ‘wanting’ infant and adult faces. Women’s mean ratings of the attractiveness of infant faces were significantly higher than men’s mean ratings. There was no difference in women’s and men’s attractiveness ratings for the adult faces (left). Men and women’s motivational salience (measured by mean viewing times) did not differ significantly for infant or adult faces (right). Error bars represent the mean +/− standard error.

Analyses were conducted using the viewing times and attractiveness ratings averaged across all exposures using SPSS. Figure 2 presents the viewing times and attractiveness ratings for the adult and infant images by participant gender. Viewing time and attractiveness ratings were transformed using log transformations to meet criteria for normality. For the adult faces, there were no significant differences between men and women in attractiveness ratings (t(69) = −1.88 p = 0.07). However, for the infant images, women rated the infants as significantly more attractive than did the men (t(69) = −2.027, p<0.05, d = 0.47). This significant difference in attractiveness ratings was not reflected in the viewing time data; viewing times were strikingly similar for the adult (t(69) = 0.46, p = 0.65) and infant stimuli t(69) = 0.17, p = 0.86). There were no differences between either the attractiveness ratings (t(69) = 0.58, p = 0.56) or viewing times (t(69) = 0.68, p = 0.68) across the adult and infant faces. No other within-gender differences were found across either the rating or viewing measure for the adult and infant faces.

**Figure 3:**
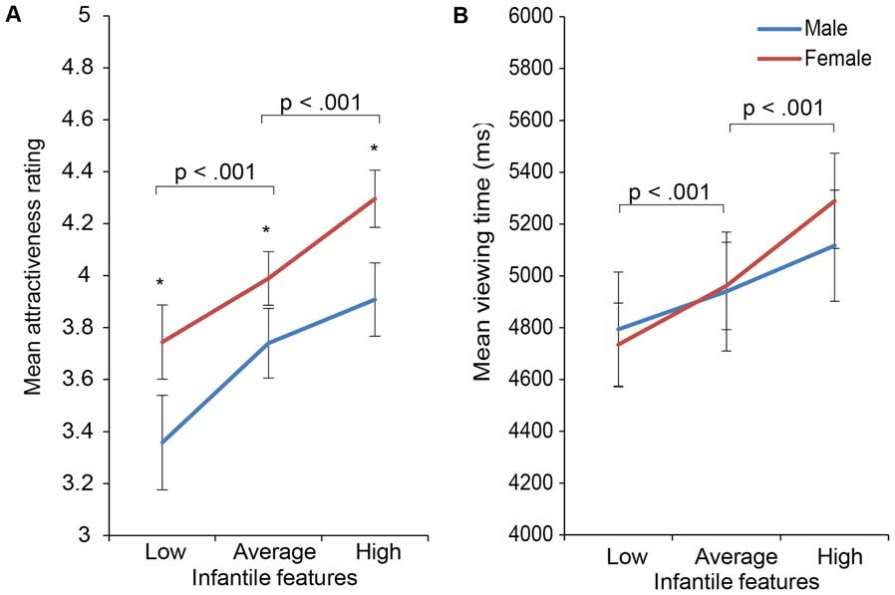
The effect of infantile features on ‘liking’ and ‘wanting’. Both men and women rated infant faces with more ‘infantile features’ as significantly more attractive than infant faces with less ‘infantile features’. Women’s overall ratings of infant attractiveness were significantly higher than men’s (left). There was a significant effect of the level of infantile features on mean viewing times, but this did not differ between men and women (right). Error bars represent mean +/− standard error. * p<0.05.

In order to further explore these differences in cuteness/attractiveness ratings to infant faces between men and women, we categorised the structure of the infant faces as high, average and low in infantile features (see Methods). We then examined the attractiveness ratings and viewing times for these three cuteness categories of infant faces by conducting a 3×2 repeated measures ANOVA with infantile features as the within-subject factor and gender as the between-subjects factor; attractiveness ratings and average viewing times were used as the outcome variables (see Figure 3).

For the ‘liking’ measures, no significant interaction between gender and infantile features category was found for the attractiveness ratings (F(1.28, 88.7) = 0.79; p = 0.4). Women did give significantly higher attractiveness ratings than men overall (F(1, 69) = 4.88, p = 0.03). Similarly, there was also a main effect of infantile feature category (F(1.3, 88.7) = 23.79, p<0.0001). Infants in the high infantile features category received higher attractiveness ratings than those in the average (t(70) = 3.9, p<0.0001) or low infantile features categories (t(70) = 5.29, p<0.0001).

For the ‘wanting’ or viewing time data, there was again no significant interaction between gender and infantile features category (F(1.5, 103.9) = 1.16, p = 0.31). In contrast to the attractiveness ratings, men and women had similar viewing times overall (F(1, 69) = 0.08, p = 0.78). Consistent with attractiveness ratings, the main effect of infantile features category was significant (F(1.5, 103.9) = 16.37, p<0.0001). Again, infants in the high infantile features category were viewed for longer than infants in the average (t(70) = 4.5, p<0.0001) or low infantile features categories (t(70) = 4.68, p<0.0001).

## Discussion

It has often been implicitly assumed that women have a greater interest in young infants than men, e.g., [43], [44]. Hedonic reactions to infants should reflect relative differences in ‘interest’. Recent insights from fundamental neuroscience have demonstrated that hedonic reactions consist of at least two partially dissociable processes of hedonic evaluation (‘liking’) and incentive salience (‘wanting’) [45]. We therefore constructed two behavioural tasks that measure attractiveness (liking) ratings, and the willingness to work, expressed in viewing times (‘wanting’). If women were simply more interested in infants than men, it would be expected that both their ‘liking’, cuteness/attractiveness ratings and their ‘wanting’, viewing times would be higher than men’s. While we did find a significant difference between men and women’s ratings of infant facial cuteness/attractiveness, we failed to find any difference in men and women’s willingness to work to view the infant faces. Critically, women were not merely rating the face stimuli as more attractive than men did: their attractiveness ratings for the adult stimuli were comparable to men’s. Men and women’s viewing times were similar for the adult faces, consistent with viewing times for the infant faces. Are men and women equally sensitive and responsive to natural variations in the degree of infantile features in infant faces? Our analysis of the cuteness/attractiveness and viewing times by category of infantile features suggests that they are. Both men and women not only rated those infants in the high infantile features as most attractive, but also worked to view those infants for the longest duration. This effect was equally apparent for men and women, suggesting that both genders are highly attuned to specific, measurable structural configurations in infant faces. While some previous studies have found that women are more able to discern experimental increased ‘cuteness’ in infant faces than men [18], [19], we found no clear cut differences in men and women’s responses to the infants varying within the natural continuum of ‘cuteness’. Interestingly, another study using natural infant stimuli within a dot probe paradigm, found that infant faces captured the attention of men and women equally well [46].

Women did provide consistently higher attractiveness ratings than men over the three categories. There are several plausible explanations for the divergence between male and female ratings of infant attractiveness. One possibility is that women were less forthright than men in rating infant attractiveness, which is potentially interesting given that women did not differ significantly from men in their mean adult attractiveness ratings. Asking participants to rate infant attractiveness is perhaps the type of sensitive question that raises social desirability issues. Another related possibility is that these measures do indeed tap into the two dissociable processes they were designed to measure: subjective appraisal or ‘liking’ and incentive salience or ‘wanting’ [45]. If this is the case, women may differ from men in their appraisal of infant stimuli but not in their motivation to work to view these stimuli. Either way, our findings underline the importance of considering both subjective appraisal *and* objective measures of behavioural responsivity to infant cues and other hedonic stimuli. Different networks of brain regions have been shown to subserve these two aspects of hedonic processing, at least where the stimuli are images of attractive men and women [28]. While our findings demonstrate adults' positive appraisal and responsiveness to infantile features, they do not imply that more attractive infants will receive more responsive care, or that less attractive infants will receive less responsive care. We deliberately tested a population with minimal experience of caring for young infants in order to investigate general responsivity to infants, and not to one’s own infant. This is, in a sense, the major limitation of this work: it remains to be seen how these experimental measures of appraisal and motivational salience translate into actual interactions with a young infant. Nonetheless, these two measures are likely to be important components in a parent's behaviour towards an infant, but the link thus far is speculative.

Our findings indicate that both men and women appraise what is colloquially described as a ‘cute’ unfamiliar infant positively, and they will work to see that infant for longer than an infant with less ‘cute’ features. This is in line with previous studies showing that ‘cuter’ infants are rated as more friendly, cheerful, and likeable [39], [47], [48], [49], [50] and are rated as more ‘adoptable’ [51].

Women’s higher ratings of infant attractiveness relative to men’s is also broadly consistent with previous work demonstrating better ‘cuteness sensitivity’ in women, e.g., [18]. That men and women show a similar level of willingness to work to see 'cute' infants speaks to the issue of the motivational salience of infant faces, an issue not tackled directly in previous studies. In light of recent findings suggesting that men are less sensitive to infant facial configuration than women (e.g., [18], [19], [31]), it is reassuring that both men and women 'want' to view infants for similar durations, suggesting a more equal interest in infants than previously thought.
